# Supplementary material for: Universal self-field critical current for thin-film superconductors
Source: Nat Commun. 2015 Aug 4;6:7820. doi: 10.1038/ncomms8820 (PMC4532791; doi:10.1038/ncomms8820)
Supplement: Supplementary Information — Supplementary Figures 1-4, Supplementary Tables 1, Supplementary Notes 1-6 and Supplementary References [file ncomms8820-s1.pdf]

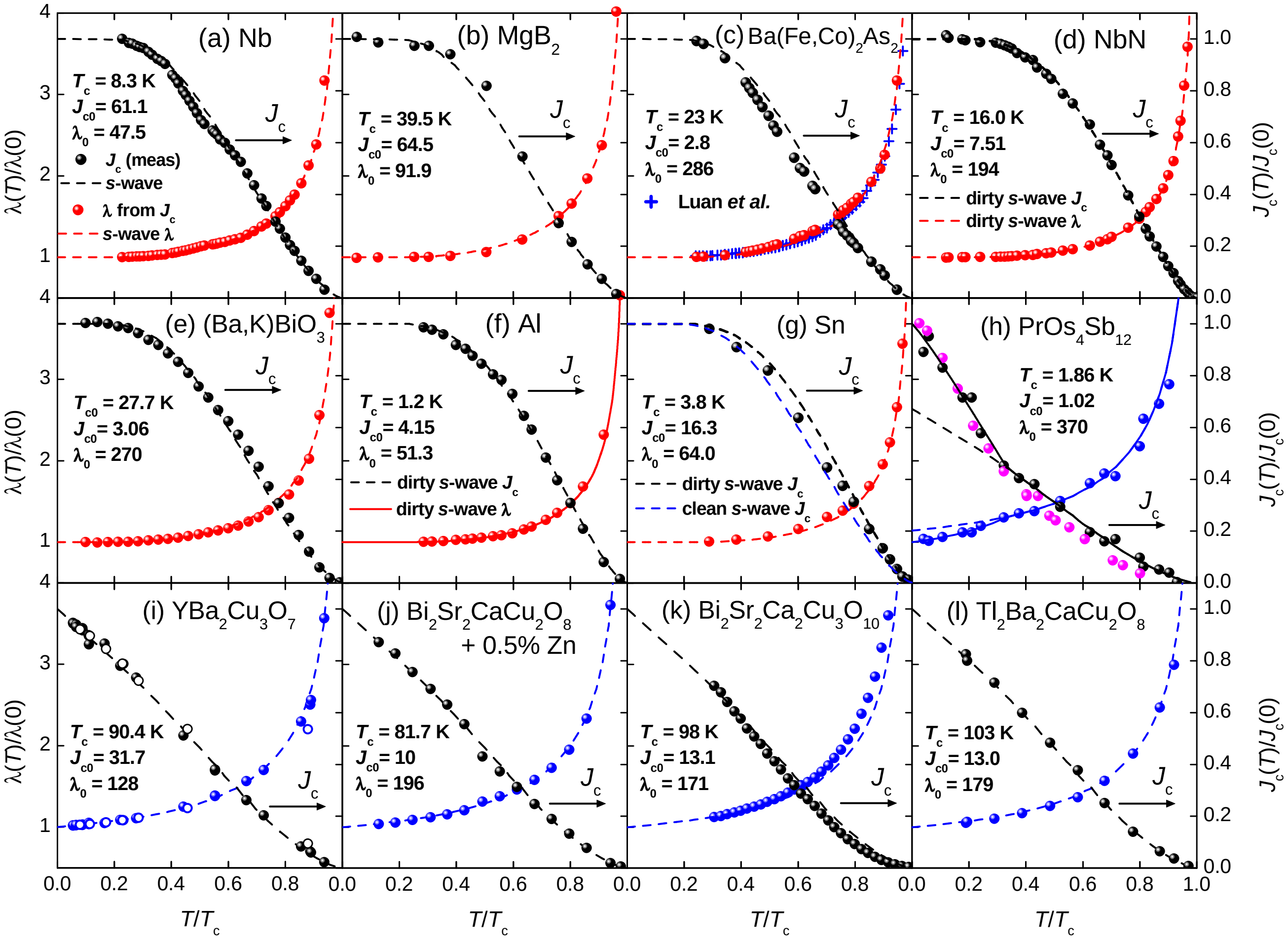

Supplementary Figure 1 | Individual plots of $\lambda(T)$ and $J_c(T, \mathrm{sf})$. The temperature dependence of the normalized penetration depth, $\lambda(T)/\lambda(0)$ (left hand scale), and critical current density, $J_c(T)/J_c(0)$ (right-hand scale), for different s-wave superconductors (red symbols and curves): (a) Nb, (b) MgB_2, (c) Ba(Fe,Co)$_2$As$_2$, (d) NbN, (e) (Ba,K)BiO_3, (f) Al and (g) Sn, and different d-wave superconductors (blue symbols and curves): (h) $PrOs_4Sb_{12}$, (i) $YBa_2Cu_3O_7$ (solid symbols) and 1% Ca-doped $YBa_2Cu_3O_7$ (open symbols), (j) $Bi_2Sr_2CaCu_2O_8$ + 5% Zn, (k) $Bi_2Sr_2Ca_2Cu_3O_{10}$ and (l) $Tl_2Ba_2CaCu_2O_8$. The dashed red curves are the s-wave weak-coupling T-dependence of $\lambda(T)$, the dashed blue curves are the d-wave weak-coupling T-dependence of $\lambda(T)$ and the dashed black curve is J_c calculated from this using equations (3) or (4). Normalization parameters, T_c, J_{c0} and $\lambda_0 = \lambda(0)$ are listed in each panel and summarized in Supplementary Table 1. In the case of (h) both J_c and λ are calculated from measurements of H_{c1} and the calculated J_c compared with measured values (magenta data points). J_{c0} is given in units of MA cm^{-2} and λ_0 in nm.

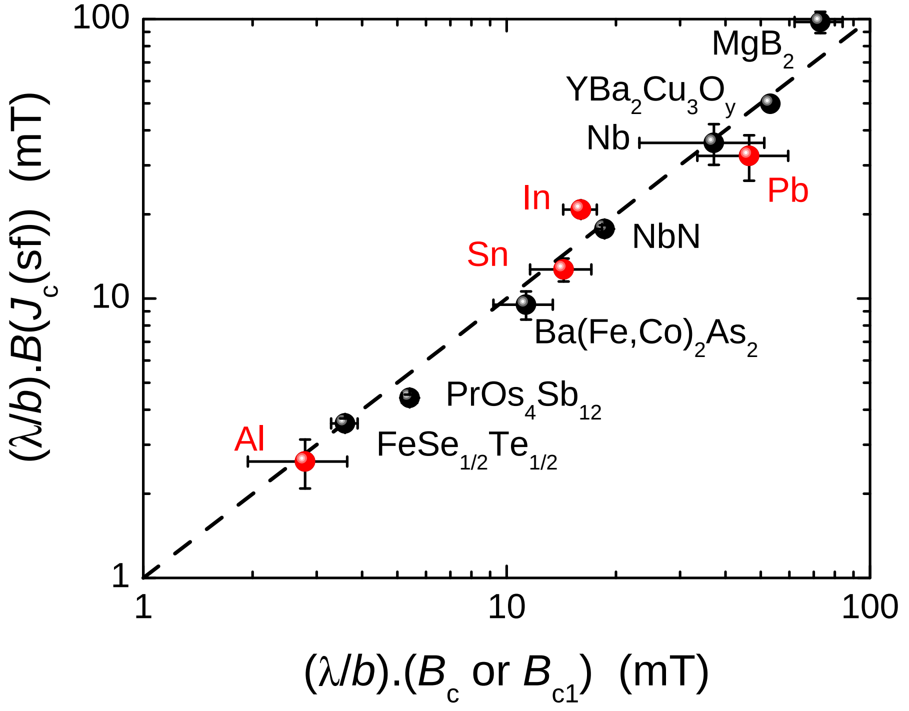

Supplementary Figure 2 | Silsbee's hypothesis confirmed. Plot of the x-component of the self field, $B_x[J_c(\text{sf})]$ on the surface of a thin-film tape versus, for type II superconductors, B_{c1} given by equation (2) (black symbols), or for type I versus B_c given by equation (1) (red symbols). Error bars reflect the uncertainty in reported values of λ; e.g. for aluminium $\lambda_0 = 50 \pm 10$ nm, as listed in Supplementary Table 1. Both axes are multiplied by (λ/b) to remove the implicit b-dependence of both of these fields.

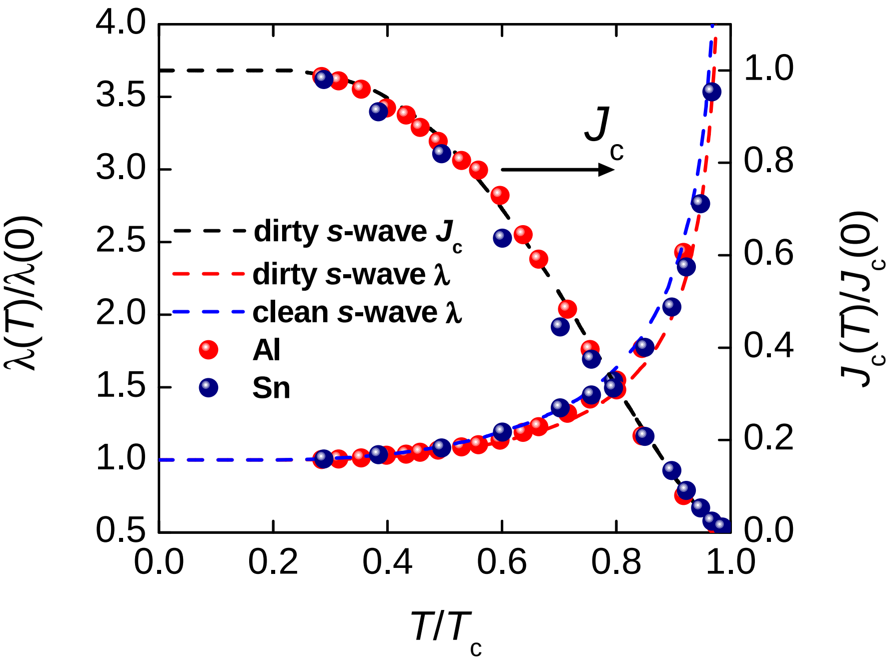

Supplementary Figure 3 | The effect of temperature dependence of κ. The effect on $\lambda(T)$ of incorporating the full T-dependence of κ using MS equation (11) for both In and Al. $\lambda(T)$ values are shifted by about half the separation of the clean- and dirty-limit curves for s-wave $\lambda(T)$.

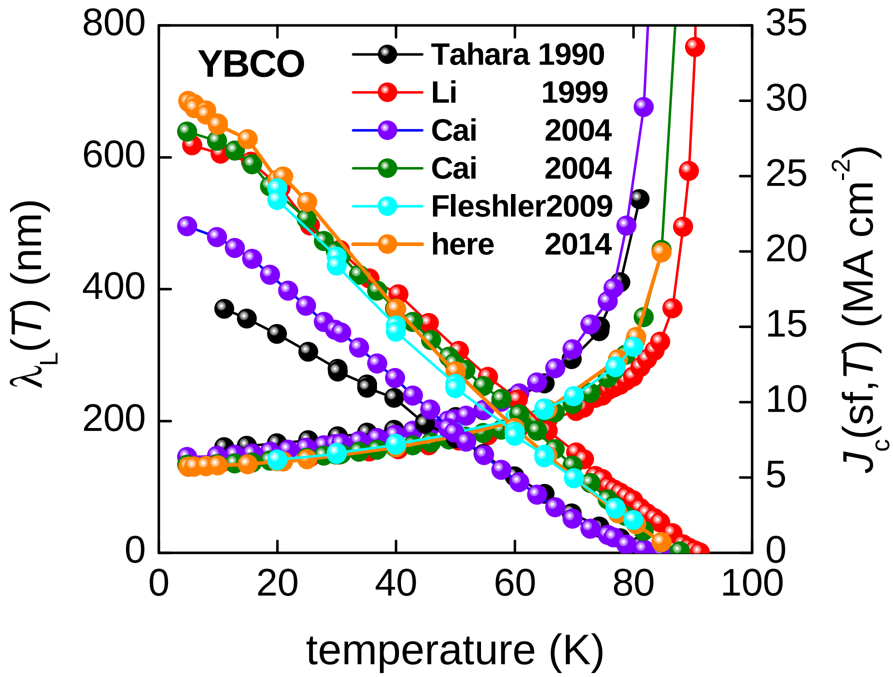

Supplementary Figure 4 | Increasing J_c over time. The T-dependence of J_c(sf) and λ calculated from equation (4) for thin films of YBCO over time [1, 2, 3, 4].

Type	Sample	T_c (K)	$2b$ (nm)	J_{c0} (MA cm^{-2})	κ	λ_0 calc (nm)	λ_0 expt (nm)	Δ_0 calc (meV)	Δ_0 expt (meV)
s-wave	Nb	8.3	20	61.1 [5]	1 [6]	47.5	47 [7] 59 [8]	1.26	1.55 [10]
							41±4 [9]		
	MgB_2	41.4	100	84.5 [11]	26 [12]	83.6	85 [13]	7.0	7.1/2.3 [15]
		41.4	100	64.5 [11]		91.7	95, 100 [14]	7.1	
	$Ba(Fe,Co)_2As_2$	23	90	2.8 [16]	90 [17]	285	270 [17] 274 [18]	2.8	3 (s-wave),
		23	90	2.5 [16]		299	307 [19]	2.9	8 (nodal) [20]
		23	90	3.1 [16]		277		3.1	
	NbN	16.0	22.5	7.51 [21]	40 [22]	194	200 [22]	2.85	2.56 [24]
							194 [23]		
		14.6	8	8.00 [25]		190		2.94	
		14.6	8	8.81 [25]		184		3.09	
		14.2	8	8.66 [26]		185		2.99	
		14.2	8	8.13 [26]		189		2.89	
	$(Ba,K)BiO_3$	27.7	150	3.06 [27]	60 [28]	270	289 [29] 270 [30]	4.46	4.5 [32] 4.3[33]
							340 [31]		
	amorph-W	4.7	50	0.35 [34]	90 [34]	550±12	-	0.71±0.02	0.66 [35]
	Rb_3C_{60}	30.5		2.15 [36]	124 [37]	319	247 [37]	3.95±0.2	3.75 [39]
							420 [38]		
	Rb_2NaC_{60}	26.3		7.0 [40]		215			
	YNi_2B_2C	15.5	600	2.12 [41]	15 [42]	158†	120 [43]	2.44±0.05	2/2.1/2.5 [44]
	Al*	1.203	89	4.15 [45]	0.03 [22]	51.3	50±10 [46]	0.21	0.179 [10]
		1.196	98	4.51 [45]		49.9	46-51 [47]	0.21	
		1.203	99	3.45 [45]		54.5	51.5 [48]	0.21	
		1.267	34	3.81 [45]		52.5		0.18	
		1.356	20	3.68 [45]		53.1		0.19	
	Sn*	3.8	50	16.3 [49]	0.23 [22]	63.3	56-68 [50]	0.62	0.593 [10]
	Pb*	7.2 [51]	47.5	52.6 [49]	0.48 [22]	55.3	39-59 [51, 52]		
	In*	3.41	100	41.3 [53]	0.11 [22]	36.5	40,38 [22, 53]	0.537	0.541 [10]
d-wave	$FeSe_{1/2}Te_{1/2}$	12.5	100	0.555 [54]	180 [55]	490	491 [55] 534 [56]	1.69	1.70 [57]
	$PrOs_4Sb_{12}$	1.86		1.02 [58]	29.7 [59, 60]	370	353 [61] 344 [62]	0.19	0.207 [62]
	$(Y,Dy)Ba_2Cu_3O_7$	90.4	850	31.7 [here]	95 [22]	128	125 [63]	16.6	16.7 [64]
	$(Nd,Er,Gd)Ba_2Cu_3O_7$	91	50	30.0 [1]	95 [22]	130	118 [65]	16.9	16.7 [64]
	$NdBa_2Cu_3O_7$	92.5	100-200	29 [2]	95 [22]	131±2		18.7±3.3	16.7 [64]
	$Bi_2Sr_2CaCu_2Zn_{0.01}O_8$	88	300-420	7.43 [66]	170 [67]	193	180 [68]	13.4	20.5, 23 [69, 70]
	$Bi_2Sr_2Ca_2Cu_3O_{10}$	92	100-150	13.5 [71]	170 [72]	160	151-155 [73]	10.8	30 [70]
	$Tl_2Ba_2CaCu_2O_8$	103	650±50	13.0 [74]	150 [75]	174	139 [76] 188 [65]	15.7	16-28 [77]
	$HgBa_2CaCu_2O_6$	118	250	10.4 [78]	123 [79]	188	145 [80] 188 [65]	16.1	32 [81]

Supplementary Table 1 | Parameters used or calculated in the analysis. References for T_c and film thickness, $2b$, are listed by the J_c values. *Type I superconductors. †using the $(\lambda/b)\tanh(b/\lambda)$ correction to MS equation (4).

Supplementary Note 1 | data for individual samples

We show in Supplementary Fig. 1 individual plots of $\lambda(T)/\lambda(0)$ and J_{cT}/J_{c0} versus T/T_c for each material in Fig. 1 of the MS. The red and blue dashed curves are the *s*- or *d*-wave T-dependence of $\lambda(T)/\lambda(0)$ [82], respectively, which are presented for comparison with the calculated values. All input or calculated parameter values are individually listed in Supplementary Table 1.

The first six examples in Supplementary Fig. 1 are *s*-wave superconductors. Starting with Nb, panel (a) shows the J_c data from Rusanov *et al.* [5], and the values of $\lambda(T)$ calculated using equation (3) with $\kappa = 1$ [6]. The dashed red curve shows the weak-coupling *s*-wave dependence. $\lambda(0)$ is found to be 47.5 nm which agrees with the observed values of 47 nm [7], 59 nm [8] and 41 ± 4 [9]. For the $\lambda(T)$ fit we used $T_c = 8.3$ K [5].

Supplementary Fig. 1(b) shows the same analysis for MgB_2 using J_c data from Zhuang *et al.* [11] and $\kappa = 26$ [12] to calculate $\lambda(T)$. The inferred value $\lambda_0 = 91.9$ nm agrees well with the range of values reported from μSR, 85 - 100 nm [13, 14]. And there is a good fit to the *s*-wave weak-coupling model except for a small divergence in the mid-temperature range which possibly arises from two gaps coexisting on distinct bands [83]. Zhuang *et al.* [11] report a second film with higher J_c still. For this we find $J_c(0) = 84.5$ MA cm^{-2} and $\lambda_0 = 83.6$ nm, again in excellent agreement with reported values.

Similarly, Supplementary Fig. 1(c) shows $\lambda(T)$ calculated for $Ba(Fe,Co)_2As_2$ using J_c data from Rall *et al.* [16] with $\kappa = 90$ [17]. The deduced value of $\lambda_0 = 286$ nm agrees well with the measured value of 270 nm [17] and there is a good fit to the *s*-wave model with the small departure probably also arising from two-gap behavior.

Data for NbN, shown in Supplementary Fig. 1(d), is based on the J_c data from Fig. 3 of Clem *et al.*, [21], $\kappa = 40$ [22]. The calculated $\lambda(T)$ is fitted to the dirty *s*-wave model (dashed red curve) which, with equation (3), is then used to calculate $J_c(T)$ shown by the dashed black curve. The fits are excellent and the calculated λ_0 value of 194 nm agrees with reported measurements by Poole *et al.* [22] (200 nm) and Komiyama *et al.* [23] (194 nm).

Supplementary Fig. 1(e) shows the analysis for $(Ba,K)BiO_3$ using the critical current data of Schweinfurth *et al.* [27] and $\kappa = 60$ [28]. We calculate $\lambda_0 = 270$ nm which is consistent with reported values 289 nm [29], 270 [30] and 340 nm [31] and we derive the value $\Delta_0 = 4.46$ meV, again very consistent with reported values of 4.5 meV [32] and 4.3 meV [33].

The results for aluminium are summarized in Supplementary Fig. 1(f). We take $\kappa = 0.03$ [22] and analyze the J_c data of Romijn *et al.* [45] who present measurements for 6 samples. The figure shows results for sample 3 but similar results are found for all samples. The value of $\lambda_0 = 51.3$ nm is in good agreement with reported values of 50 ± 10 nm [46], and the dashed curves are the dirty s-wave calculation. For the other samples we find λ_0 values of 49.9 nm (#1), 54.5 nm (#2), 52.5 nm (#4) and 53.1 nm (#5). Supplementary Fig. 1(g) shows that similar results are also found for tin. J_c data are from Hunt [49] and we obtain $\lambda_0 = 63.3$ nm in excellent agreement with the range 56 - 68 nm reported by Peabody [50]. The $\lambda(T)$ fit seems best when intermediate between clean and dirty s-wave.

We perform the analysis of J_c data for $FeSe_{0.5}Te_{0.5}$ [54] using $\kappa = 180$ [55] (not shown in the figure). The fit is excellent with $\lambda_0 = 490$ nm in good agreement with the reported λ_0 values of 491 nm [55] and 534 nm [56]. However, the situation is complicated by the likely presence of multiple gaps of differing symmetry [84] and the data of Belingeri [54] does not extend to low enough temperature to settle the issue.

Turning now to the class of d-wave superconductors, in Supplementary Fig. 1(h) for $PrOs_4Sb_{12}$ we used a slightly different approach. From reported data for H_{c1} [58] we calculated both $J_c(T)$ and $\lambda(T)$ from equation (4) using $\kappa = 29.66$. Each of these parameters reveals a transition to a second superconducting phase below 0.6 K which results in an additional reduction of $\lambda(T)$. With these coexisting phases we simply added d-wave superfluid densities to achieve a fit to the data. Most importantly for our central thesis, the measured $J_c(T)$ (from remanent magnetization measurements [58]) shows an enhancement below 0.65 K that almost exactly mirrors our calculated $J_c(T)$.

The figure is completed by several high-T_c cuprates. In Supplementary Fig. 1(i) for $YBa_2Cu_3O_7$ (solid data points) we used our own epitaxial MOD-deposited 2G tapes with lithographically-etched conductance bridges and silver electrodes. The overall composition was $(YDy_{0.5})Ba_2Cu_3O_7$ and film thickness $\approx 0.8\mu$m. We used $\kappa = 95$ [22] and the calculated $\lambda_0 = 128$ nm is an excellent match with the single-crystal value from μSR, $\lambda_0 = 125$ nm [63] which also concurs with polycrystalline data [65] and infrared data [65, 85]. This indicates maximum practical self-field performance of these 2G tapes. Open data points are for 1% Ca-doped $YBa_2Cu_3O_7$ with $T_c = 88$ K. This also shows an excellent fit with $\lambda_0 = 144$ nm, the increase being due to impurity scattering and/or overdoping where the superfluid density is known to fall [86]. The combined data shown in Supplementary Fig. 1(i) for YBCO nicely

shows the canonical d-wave inflexion in the T-dependence of $J_c(T)$ with negative curvature at low-T and positive curvature at high-T.

In Supplementary Fig. 1(j) for $Bi_2Sr_2CaCu_2O_8$ + 0.5% Zn we use the J_c data of Wagner *et al.* [66] which extends to quite low temperature. Together with $\kappa = 170$ (as an average of many different reports e.g. see [67]) we find a good d-wave fit to $\lambda(T)$ and the $J_c(T)$ data again shows the distinctive d-wave inflexion. The deduced $\lambda_0 = 193$ nm agrees quite well with the measured value of 180 nm [68]. It is expected to be a little high due to the suppression of superfluid density by the small fraction of Zn impurity scatterers.

In contrast Supplementary Fig. 1(k) for $Bi_2Sr_2Ca_2Cu_3O_{10}$ shows $\lambda(T)$ rises above the d-wave fit at higher T and this is likely due to the presence of intergrowths of $Bi_2Sr_2CaCu_2O_8$. Despite an onset at 110 K the rather low T_c of 92 K [71] for these films would appear to support this. $\lambda(T)$ and the superfluid density are very sensitive to such intergrowth effects and this evidently plays through into the detailed behavior of $J_c(T)$. Here we used J_c data of Hänisch [71] along with $\kappa = 170$ [72].

Finally, in Supplementary Fig. 1(ℓ) for $Tl_2Ba_2CaCu_2O_8$ the J_c data from Holstein [74] and $\kappa = 150$ [75] gives a good d-wave fit across the full T-range for both $\lambda(T)$ and $J_c(T)$ indicating a good quality single-phase film. The calculated value $\lambda_0 = 174$ nm is in reasonable agreement with the experimental values of 139 nm [76] and 188 nm [65].

Supplementary Note 2 | Silsbee's hypothesis demonstrated

We now show explicitly that, for $b \leq \lambda$, the field on the flat face of a thin film conductor when $J = J_c(\mathrm{sf})$ is indeed the critical field as proposed by Silsbee [87]. From equation (12) of Brojeny and Clem [88] the x-component of B at $y = \pm b$ for a uniform current density is $-\mu_0 b J_c$. Because this field is b-dependent we multiply by the factor (λ/b) to remove this dependence. Similarly, as noted in equation (11) of the MS B_{c1} scales as (b/λ) when $b < \lambda$, so again we remove this b-dependence by multiplying by (λ/b). Thus, for type II superconductors $(\lambda/b) \times B_x(J_c)$ is plotted in Supplementary Fig. 2 versus $(\lambda/b) \times B_{c1}$ given by equation (2) (black symbols), or for type I superconductors versus $(\lambda/b) \times B_c$ given by equation (1) (red symbols). The correspondence is excellent over nearly two orders of magnitude, and since both axes are multiplied by the same factor this confirms Silsbee's hypothesis.

Supplementary Note 3 | Temperature dependence of κ

We now consider the effect of the until-now ignored T-dependence of κ. For this we adopt the weak-coupling s-wave T-dependence of $\lambda(T) \times \Delta(T)$ [82]. The result is shown in Supplementary Fig. 3 for the type I superconductors Al and Sn. Without this correction both show a good fit to the dirty s-wave model (see Supplementary Fig. 1(e) and (f)). With the correction the calculated $\lambda(T)$ shifts towards the clean-limit behaviour. The effect is small, amounting to half the shift between the dirty- and clean-limits, and we conclude that our initial approximation of a constant κ is not unreasonable.

Supplementary Note 4 | Further comments on pinning

A key conclusion of our work is that J_c(sf) is fundamentally limited by λ and not by pinning when the thickness is comparable to λ. As noted, this challenges a prevailing view [89, 90, 91]. In addition to the literature examples discussed in the MS which support our view we note here further examples where marked increases in pinning, and associated improvement in in-field J_c, are demonstrated while there is no observed change in J_c(sf). These include MacManus-Driscoll, *et al.* [92], Strickland *et al.* [93], Birlick *et al.* [94], Lu *et al.* [95] and Haruta *et al.* [96].

The remaining exceptional example is that of Dinner *et al.* [91] in which the introduction of nanopores in Nb raises J_c(sf) by a factor of 30. But here the film thickness is of the order of the coherence length, ξ, and the nanopore spacing is just a few times ξ. The nanopores therefore represent radical modification and it is not impossible that ion milling through the Al_2O_3 capping layer results in the formation of Nb_3Al with consequent increase in J_c(sf) due to a smaller λ value. In any case, this enhanced J_c(sf) is still less than that reported by Rusanov *et al.* [5] at the same temperature, as presented in Supplementary Fig. 1(a) and Supplementary Table 1. It thus remains to be demonstrated that pinning centres created by any means actually increase J_c(sf).

Supplementary Note 5 | London versus Ginzburg-Landau depairing currents

We note that equation (3) is identical to the London depairing current density J_d^L [97] and a criticism could arise from the fact that it is actually larger than the so-called Ginzburg-Landau pairbreaking current density [97]

$$J_d^{GL} = \frac{\phi_0 \kappa}{3\sqrt{3}\pi \mu_0 \lambda^3(T)}, \tag{1}$$

by a factor $(3/2)^{3/2} = 1.84$. Were one to adopt this as the fundamental J_c limit then after taking the cube root λ values calculated in Fig. 1(a) of the MS for type I superconductors will be reduced by a factor 0.82. Actually, this could possibly be accommodated in the figure. On the other hand the relative merits of the two depairing limits have not been tested and, moreover, the theoretical calculation of J_d^{GL} requires an approximation in neglecting the gradient term [97]. For the time being equation (3) seems to be well satisfied and we retain it as an effective measure of J_c for this class of superconductor.

Supplementary Note 6 | Increase in J_c over time for YBCO

Finally, the improvement in J_c(sf) over time for $YBa_2Cu_3O_{7-\delta}$ is summarized in Supplementary Fig. 4 [1, 2, 3, 4]. Notably all the recent data return values of λ_0 close to the observed value and all data retain essentially the same T-dependent shape. Even the early data from 1990 show the distinctive d-wave linear-T temperature dependence several years before this particular symmetry was established. Some however, display a possible T^2 dependence at low-T more characteristic of the superfluid density in a dirty d-wave scenario. This suggests a critical test of our hypothesis, namely, to establish, by systematic measurements of J_c(sf), a crossover from linear-T to T^2 behavior with increasing impurity scattering. This work is proceeding.

Supplementary References

---

[1] Cai, C., Holzapfel, B., Hänisch, J., Fernández, L., & Schultz, L. High critical current density and its field dependence in mixed rare earth (Nd,Eu,Gd)$Ba_2Cu_3O_{7-\delta}$ thin films. *Appl. Phys. Lett.* **84**, 377-379 (2004).

[2] Li, Y. & Tanabe, K. Influence of deposition parameters on superconductivity and surface morphology of $NdBa_2Cu_3O_{7-\delta}$ films grown by pulsed laser deposition with a single crystal target. *Physica* C **324**, 198-210 (1999).

[3] Tahara, S., Anlage, S.M., Halbritter, J., Eom, C.B., Fork, D.K., Geballe, T.H. & Beasley, M.R. Critical currents, pinning, and edge barriers in narrow $YBa_2Cu_3O_{7-y}$ thin films. *Phys. Rev.* B **41**, 11203-11208 (1990).

[4] Fleshler, S., *et al.* Scale-up of 2G wire manufacturing at American Superconductor Corporation. *Physica* C **469**, 1316-1321 (2009).

[5] Rusanov, A.Yu., Hesselberth, M.B.S. & Aarts, J. Depairing currents in superconducting films of Nb and amorphous MoGe. *Phys. Rev. B* **70**, 024510 (2004).

[6] Kim, D.H. *et al.* Resistive measurement of the temperature dependence of the penetration depth of Nb in Nb/AlOx/Nb Josephson junctions. *J. Appl. Phys.* **75**, 8163-8167 (1994).

[7] Maxfield, B.W. & McLean, W.L. Superconducting penetration depth of niobium. *Phys. Rev. A* **139**, 1515-1522 (1965).

[8] Masuradze, A., *et al.* Evidence for Cooper pair diffraction on the vortex lattice of superconducting niobium. *Phys. Rev.* B **88**, 140509(R) (2013).

[9] Felcher, G.P., Kampwirth, R.T., Gray, K.E., & Felici, R. Polarized-neutron reflections: a new technique used to measure the magnetic field penetration depth in superconducting niobium. *Phys. Rev. Lett.* **52**, 1539 (1984).

[10] Wolf, E.L., Principles of Electron Tunneling Spectroscopy (Oxford University Press, New York, 1985), pp. 524-529.

[11] Zhuang, C.G., Meng, S., Zhang, C.Y., Feng, Q.R., Gan, Z.Z., Yang, H., Jia, Y., Wen, H.H., & Xi, X.X. Ultrahigh current-carrying capability in clean MgB_2 films. *J. Appl. Phys.* **104**, 013924 (2008).

[12] Buzea, C & Yamashita, T. Review of the superconducting properties of MgB_2. *Supercond.*

Sci. Technol. **14**, R115-R146 (2001).

[13] Panagopoulos, C., Rainford, B. D., Xiang, T., Scott, C. A., Kambara, M. & Ir, I. H. Penetration depth measurements in MgB_2: evidence for unconventional superconductivity. *Phys. Rev.* B **64** 094514 (2001).

[14] Niedermayer, Ch., Bernhard, C., Holden, T., Kremer, R.K., & Ahn, K. Muon spin relaxation study of the magnetic penetration depth in MgB_2. *Phys. Rev. B* **65**, 094512 (2002).

[15] Iavarone, M. *et al.* Directional scanning tunneling spectroscopy in MgB_2. *Physica* C **385** 215 (2003).

[16] Rall, D. *et al.* Critical current densities in ultrathin $Ba(Fe,Co)_2As_2$ microbridges. *Phys. Rev. B* **83**, 134514 (2011).

[17] Gordon R.T. *et al.* Doping evolution of the absolute value of the London penetration depth and superfluid density in single crystals of $Ba(Fe_{1-x}Co_x)_2As_2$. *Phys. Rev. B* **82**, 054507 (2010).

[18] Luan, L., *et al.* Local measurement of the superfluid density in the pnictide superconductor $Ba(Fe_{1-x}Co_x)_2As_2$ across the superconducting dome. *Phys. Rev. Lett.* **106**, 067001 (2011).

[19] Williams, T.J., *et al.* Superfluid density and field-induced magnetism in $Ba(Fe_{1-x}Co_x)_2As_2$ and $Sr(Fe_{1-x}Co_x)_2As_2$ measured with muon spin relaxation. *Phys. Rev. B* **84**, 094512 (2010).

[20] Fischer, T. *et al.* Highly anisotropic energy gap in superconducting $Ba(Fe_{0.9}Co_{0.1})_2As_2$ from optical conductivity measurements. *Phys. Rev.* B **82**, 224507 (2010).

[21] Clem, J.R., Bumble, B., Raider, S.I., Gallagher, W.J. & Shih, Y.C. Ambegaokar-Baratoff-Ginzburg-Landau crossover effects on the critical current density of granular superconductors. *Phys. Rev. B* **35**, 6637-6642 (1987).

[22] Poole, C.P., Farach, H.A., Creswick, R.J., Prozorov, R. *Superconductivity* Ch. 12, p. 343 (Academic Press, London, 2007).

[23] Komiyama, B., Wang, Z., & Tonouchi, M. Penetration depth measurements of single-crystal NbN films at millimeter-wave region. *Appl. Phys. Lett.* **68**, 562-563 (1996).

[24] Kihlstrom, K.E., Simon, R.W. & Wolf, S.A., Tunneling $\alpha^2 F(\omega)$ from sputtered thin-film NbN. *Phys. Rev.* B **32**, 1843 (1985).

[25] Bartolf, H., Engel, A., Schilling, A., Ilin, K. & Siegel, M. Fabrication of metallic structures with lateral dimensions less than 15 nm and $J_c(T)$-measurements in NbN micro- and nanobridges. *Physica* C **468**, 793-796 (2008).

[26] Engel, A., Bartolf, H., Schilling, A., Il'in, K., Siegel, M., Semenov, A. & Hubers, H.-W.

Temperature- and field-dependence of critical currents in NbN microbridges. *J. Phys. Conf. Series* **97**, 012152 (2008).

[27] Schweinfurth, R. A., Platt, C. E., Teepe, M. R. & van Harlingen, D. J. Electrical and magnetic properties of laser-deposited $Ba_{1-x}K_xBiO_3$ thin films. *Appl. Phys. Lett.* **61**, 480-482 (1992).

[28] Barilo, S. N., Shiryaev, S. V., Gatalskaya, V. I., Lynn, J. W., Baran, M., Szymczak, H., Szymczak, R. & Dew-Hughes, D. Scaling of magnetization and some basic parameters of $Ba_{1-x}K_xBiO_3$ superconductors near T_c. *Phys. Rev.* **58**, 12355 (1998).

[29] Mosley, W. D., Liu, J.Z., Matsushita, A., Lee, Y. P., Klavins, P. & Shelton, R. N. Preparation and superconductivity of $Ba_{1-x}K_xBiO_3$ single crystals.

[30] Uchida, T., Nakamura, S., Suzuki, N., Nagata, Y., Mosley, W. D., Lan, M. D., Klavins, P. & Shelton, R. N. Effect of growth conditions on the superconductivity of $Ba_{1-x}K_xBiO_3$ crystals. *Physica* C **215**, 350-358 (1993).

[31] Ansaldo, E. J., Wang, Z. R., Cho, J., Johnston, D. C. & Riseman, T. M. Magnetic penetration depth of $Ba_{0.625}K_{0.375}BiO_3$. *Physica* C **185-189**, 1889-1890 (1991).

[32] Huang, Q., Zasadzinski, J. F., Tralshawala, N., Gray, K. E., Hinks, D. G., Peng, J. L. & Greene, R. L. Tunnelling evidence for predominantly electron-phonon coupling in superconducting $Ba_{1-x}K_xBiO_3$ and $Nd_{2-x}Ce_xCuO_{4-y}$. *Nature* **347**, 369-372 (1990).

[33] Kosugi, M., Akimitsu, J., Uchida, T., Furuya, M., Nagata, Y. & Ekino, T. Tunneling studies on $Ba_{1-x}K_xBiO_3$ single crystals with T_c = 15-30 K. *Physica* C **229**, 389-395 (1994).

[34] Sun, Y., Wang, J., Zhao, W., Tian, M., Singh, M. & Chan, M.H.W. Voltage-current properties of superconducting amorphous tungsten nanostrips. *Scientific Rep.* **3**, 2307 (2013).

[35] Guillamón, I., Suderow, H., Vieira, S., Fernández-Pacheco, A., Sesé, J., Córdoba, R., De Teresa, J.M., & Ibarra, M.R. Nanoscale superconducting properties of amorphous W-based deposits grown with a focused-ion-beam. *New J. Phys.* **10**, 093005 (2008).

[36] Tai, M.F., Chang, G.F. & Lee, M. W. Vortex-lattice melting in superconducting fullerene Rb_3C_{60}. *Phys. Rev.* B **52**, 1176-1180 (1995).

[37] Sparn, G., Thompson, J.D., Whetten, R.L., Huang, S.-M., Kaner, R.B., Diederich, F., G. Grüner & Holczer, K. Pressure and field dependence of superconductivity in Rb_3C_{60}. *Phys. Rev. Lett.* **68**, 1228-1231 (1992).

[38] Uemura, Y.J., Keren, A., Le, L.P., Luke, G.M., Wu, W.D., Tsai, J.S., Tanigaki, K., Holczer, K., Donovan, S. & Whetten, R.L. System dependence of the magnetic-field penetration depth

in C_{60} superconductors. *Physica* C **235-240**, 2501-2502 (1994).

[39] Degiorgi, L., Wachter, P., Grüner, G., Huang, S.-M., Wiley, J., & Kaner, R. B., Optical response of the superconducting state of K_3C_{60} and Rb_3C_{60}, *Phys. Rev. Lett.* **69**, 2987-2990 (1992).

[40] Glenis, S., Cooke, S., Chen X. & Labes, M.M. Superconducting properties of alkali doped C_{60} prepared by precipitation from liquid methylamine or ammonia. *Mol. Cryst. Liq. Cryst.* **284**, 139-147 (1996).

[41] Wimbush, S.C., Schultz, L. & Holzapfel, B. Critical current in YNi_2B_2C and $HoNi_2B_2C$ thin films, *Physica C* **388-389**, 191-192 (2003).

[42] Hilscher, G. & Michor, H. Superconductivity and magnetism in quaternary borocarbides, in *Studies on High Temperature Superconductors*, ed. by A.V. Narlikar **28** (New York: Nova Science, 1999) p. 241.

[43] Canepa, F., Manfrinetti, P., Palenzona, A., Cimberle, M.R., Giannini, E., Marrè, D. & Putti, M. Magnetic study of the superconducting phase YNi_2B_2C. *Il Nuovo Cimento* **16** D, 1857 (1996).

[44] Lu, X., Park, W.K., Yeo, S., Oh, K.-H., Lee, S.-I., Budko, S.L., Canfield, P.C., & Greene, L.H. Superconducting order parameter in nonmagnetic borocarbides RNi_2B_2C (R=Y,Lu) probed by point-contact Andreev reflection spectroscopy. *Phys. Rev.* B **83**, 104519 (2011).

[45] Romijn, J., Klapwijk, T.M., Renne M.J. & Mooij, J.E. Critical pair-breaking current in superconducting aluminum strips far below T_c. *Phys. Rev. B* **26**, 3648-3655 (1982).

[46] Prozorov, R. *et al.* Measurements of the absolute value of the penetration depth in high-T_c superconductors using a low-T_c superconductive coating. *Appl. Phys. Lett.* **77**, 4202-4204 (2000).

[47] Doezema, R.E., Whitmore, S.C., & Huffaker, J.N. Quasiparticle magnetospectroscopy and penetration depth anisotropy in superconducting aluminum. *Phys. Rev. B* **34**, 4614-4621 (1986).

[48] Biondi, M.A., & Garfunkel, M.P. Millimeter wave absorption in superconducting aluminum. II. Calculation of the skin depth. *Phys. Rev.* **116**, 862-867 (1982).

[49] Hunt, T.K. Critical-current behavior in narrow thin-film superconductors. *Phys. Rev.* **151**, 325-327 (1966).

[50] Peabody, G.E. & Meservey, R. Magnetic flux penetration into superconducting thin films.

Phys. Rev. B **6**, 2579-2595 (1972).

[51] Nutley, M.P., Boothroyd, A.T., Staddon, C.R., D. Paul, D.McK. & Penfold, J. Magnetic-induction profile in a type-I superconductor by polarized-neutron reflectometry. *Phys. Rev.* B **49**, 15789-15798 (1994).

[52] Brisbois, J., Raes, B., Van de Vondel, J., Moshchalkov, V.V. & Silhanek, A.V. Determination of the magnetic penetration depth in a superconducting Pb film. *J. Appl. Phys.* **115**, 103906 (2014).

[53] Neumann, L.G. & Kao, Y.H. Temperature dependence of the critical current in one-dimensional indium strips. *J. Low Temp. Phys.* **48**, 321-330 (1982).

[54] Bellingeri, E. *et al.* Strong vortex pinning in $FeSe_{0.5}Te_{0.5}$ epitaxial thin film. *Appl. Phys. Lett.* **100**, 082601 (2012).

[55] Bendele, M., *et al.* Anisotropic superconducting properties of single-crystalline $FeSe_{0.5}Te_{0.5}$. *Phys. Rev. B* **81**, 224520 (2010).

[56] Biswas, P.K., Balakrishnan, G., Paul, D.M., Tomy, C.V., Lees, M.R., & Hillier, A.D. Muon-spin-spectroscopy study of the penetration depth of $FeSe_{0.5}Te_{0.5}$. *Phys. Rev.* B **81**, 092510 (2010).

[57] Ukita, R., Sugimoto, A., & Ekino, T. Scanning tunneling microscopy and break-junction spectroscopy on the superconducting Fe(Se,Te) single crystal *Physica* C **484**, 22-26 (2013).

[58] Cichorek T., Mota, A.C., Steglich, F., Frederick, N.A., Yuhasz, W.M. & Maple, M.B. Pronounced enhancement of the lower critical field and critical current deep in the superconducting state of $PrOs_4Sb_{12}$. *Phys. Rev. Lett.* **94**, 107002 (2005).

[59] MacLaughlin, D.E. *et al.*, Muon spin relaxation and isotropic pairing in superconducting $PrOs_4Sb_{12}$. *Phys. Rev. Lett.* **89**, 157001 (2002).

[60] Bauer, E.D., Frederick, N.A., Ho, P.-C., Zapf, V.S. & Maple, M.B. Superconductivity and heavy fermion behavior in $PrOs_4Sb_{12}$. *Phys. Rev. B* **65**, 100506(R) (2002).

[61] Shu, L., MacLaughlin, D. E., Beyermann, W. P., Heffner, R. H., Morris, G. D., Bernal, O. O., Callaghan, F. D., Sonier, J. E., Yuhasz, W. M., Frederick, N. A., & Maple, M. B. Penetration depth, multiband superconductivity, and absence of muon-induced perturbation in superconducting $PrOs_4Sb_{12}$. *Phys. Rev.* B **79**, 174511 (2009).

[62] Chia, E.E.M., Salamon, M.B., Sugawara, H., & Sato, H. Probing the superconducting gap symmetry of $PrOs_4Sb_{12}$: a penetration depth study. *Phys. Rev. Lett.* **91**, 247003 (2003).

[63] Sonier, J.E. *et al.* Hole-doping dependence of the magnetic penetration depth and vortex core size in $YBa_2Cu_3O_y$: Evidence for stripe correlations near 1/8 hole doping. *Phys. Rev. B* **76**, 134518 (2007).

[64] Dagan, Y., Krupke, R., & Deutscher, G. Determination of the superconducting gap in $YBa_2Cu_3O_{7-\delta}$ by tunneling experiments under magnetic fields. *Phys. Rev.* B **62**, 146-149 (2000).

[65] Tallon, J.L., Bernhard, C. Binninger, U., Hofer, A., Williams, G.V.M., Ansaldo, E.J., Budnick, J.I. & Niedermayer, Ch. In-plane anisotropy of the penetration depth due to superconductivity on the CuO chains in $YBa_2Cu_3O_{7-\delta}$, $Y_2Ba_4Cu_7O_{15-\delta}$ and $YBa_2Cu_4O_8$. *Phys. Rev. Lett.* **74**, 1008-1011 (1995)

[66] Wagner, P., Hillmer, F., Frey, U., & Adrian, H. Thermally activated flux movement and critical transport current density in epitaxial $Bi_2Sr_2CaCu_2O_{8+\delta}$ films. *Phys. Rev. B* **49**, 13184-13192 (1994).

[67] Ri, H.-C., Gross, R., Gollnik, F., Beck, A., Huebener, R.P., Wagner P. & Adrian, H. Nernst, Seebeck, and Hall effects in the mixed state of $YBa_2Cu_3O_{7-\delta}$ and $Bi_2Sr_2CaCu_2O_{8+x}$ thin films: A comparative study. *Phys. Rev. B* **50**, 3312-3329 (1994).

[68] Mosqueira, J. *et al.* Thermal fluctuation effects on the magnetization above and below the superconducting transition in $Bi_2Sr_2CaCu_2O_8$ crystals in the weak magnetic field limit. *Phys. Rev. B* **53**, 15272-15280 (1996).

[69] Suzuki, A., & Suzuki, M. Tunneling studies of pseudo-gap in $Bi_2Sr_2CaCu_{1.94}Zn_{0.06}O_{8-y}$. *Physica* C **378-381** 306-310 (2002).

[70] Hudákova, N., Samuely, P., Szabó, P., Plechácek, V., Knízek, K., & Sedmidubskyy, D. Scaling of the superconducting order parameter in Bi cuprates with T_{c}. *Physica* C **246**, 163-168 (1995).

[71] Hänisch, J., Attenberger, A., Holzapfel, B., & Schultz, L. Electrical transport properties of $Bi_2Sr_2Ca_2Cu_3O_{10+\delta}$ thin film [001] tilt grain boundaries. *Phys. Rev. B* **65**, 052507 (2002).

[72] Li, Q., Suenaga, M., Gohng, J., Finnemore, D.K., Hikata, T., & Sato, K. Reversible magnetic properties of c-axis-oriented superconducting $Bi_2Sr_2Ca_2Cu_3O_{10+\delta}$. *Phys. Rev. B* **46**, 3195-3198 (1992).

[73] Weigand, M., Eisterer, M., Giannini, E. & Weber, H.W. Mixed state properties of $Bi_2Sr_2Ca_2Cu_3O_{10+\delta}$ single crystals before and after neutron irradiation. *Phys. Rev. B* **81**, 014516 (2010).

[74] Holstein, W.L., Wilker, C., Laubacher, D.B., Face, D.W., Pang, P., Warrington, M.S., Carter, C.F. & Parisi, L.A. Critical current density and resistivity measurements for long patterned lines in $Tl_2Ba_2CaCu_2O_8$ thin films. *J. Appl. Phys.* **74**, 1426-1430 (1993).

[75] Kim, H.-J., Chowdhury, P., Jo, I.-S. & Lee, S.-I. Equilibrium magnetization of $Tl_2Ba_2CaCu_2O_{8+\delta}$ single crystals. *Phys. Rev. B* **66**, 134508 (2002).

[76] Moonen, J.T. & Brom, H.B. Vortex-mobility determined NMR properties in highly anisotropic high-T_c superconductors II. Experiment on $Tl_2Ba_2CaCu_2O_8$. *Physica C* **244**, 10-20 (1995).

[77] Huang, Q., Zasadzinski, J.F., Gray, K.E., Bukowski, E.D., & Ginsberg, D.M. Point-contact tunneling study of the normal and superconducting states of $Tl_2Ba_2CaCu_2O_x$. *Physica* C **161**, 141-144 (1989).

[78] Krusin-Elbaum, L., Tsuei, C.C & Gupta, A. High current densities above 100 K in the high-temperature superconductor $HgBa_2CaCu_2O_{6+\delta}$. *Nature* **373**, 679-681 (1995).

[79] Pužniak, R., Usami, R., Isawa, K., & Yamauchi, H. Superconducting-state thermodynamic parameters and anisotropy of $HgBa_2Ca_{n-1}Cu_nO_y$ by reversible magnetization measurements. *Phys. Rev. B* **52**, 3756-3764 (1995).

[80] Thompson, J.R., Ossandon, J.G., Christen, D.K., Paranthaman, M., Specht, E.D. & Kim, Y.C. Comparative study of the characteristic length scales and fields of Hg-based high-T_c superconductors. *Phys. Rev. B* **54**, 7505-7511 (1996).

[81] Sacuto, A., Cayssol, J., Monod, P., & Colson, D. Electronic Raman scattering on the underdoped $HgBa_2Ca_2Cu_3O_{8+d}$ high-T_c superconductor: The symmetry of the order parameter. *Phys. Rev.* B **61** 7122-29 (2000).

[82] Won, H. & Maki, K. *d*-wave superconductor as a model of high-T_c superconductors. *Phys. Rev. B* **49**, 1397-1402 (1994).

[83] Kogan, V.G., Martin C. & Prozorov, R. Superfluid density and specific heat within a self-consistent scheme for a two-band superconductor. *Phys. Rev. B* **80**, 014507 (2009).

[84] Homes, C. C., Akrap, A., Wen, J. S., Xu, Z. J., Lin, Z. W., Li, Q., & Gu, G. D. Electronic correlations and unusual superconducting response in the optical properties of the iron chalcogenide $FeTe_{0.55}Se_{0.45}$. *Phys. Rev.* B **81**, 180508(R) (2010).

[85] Yu, L., *et al.* Evidence for two separate energy gaps in underdoped high-temperature cuprate superconductors from broadband infrared ellipsometry. *Phys. Rev. Lett.* **100**, 177004 (2008).

[86] Bernhard, C., Tallon, J.L., Blasius,, Th., Golnik, A., & Niedermayer, Ch. Anomalous peak

in the superconducting condensate density of cuprate high-T_c superconductors at a unique doping state. *Phys. Rev. Lett.* **86**, 1614 (2001).

[87] Silsbee, F.B. Note on electrical conduction in metals at low temperatures. *J. Franklin Inst.* **184**, 111 (1917).

[88] Brojeny, A.B. & Clem, J.R., Self-field effects upon critical current density of flat superconducting strips. *Supercond. Sci. Technol.* **18**, 888-895 (2005).

[89] Brandt, E.H. & Indenbom, M., Type-II-superconductor strip with current in a perpendicular magnetic field. *Phys. Rev. B* **48**, 12893-12906 (1993).

[90] Stejic, G., Gurevich, A., Kadyrov, E., Christen, D., Joynt, R., & Larbalestier, D. C., Effect of geometry on the critical currents of thin films. *Phys. Rev.* B **49**, 1274-1288 (1994).

[91] Dinner, R.B., Robinson, A.P., Wimbush, S.C., MacManus-Driscoll, J.L., & Blamire, M.G. Depairing critical current achieved in superconducting thin films with through-thickness arrays of artificial pinning centers. *Supercond. Sci. Technol.* **24** 055017 (2011).

[92] MacManus-Driscoll, J.L. *et al.* Strongly enhanced current densities in superconducting coated conductors of $YBa_2Cu_3O_{7x}$ + $BaZrO_3$. *Nature Materials* **3** 439-443 (2004).

[93] Strickland, N.M., Talantsev, E.F., Xia, J.A., Long, N.J., Rupich, M.W., Li, X., & Zhang, W. Flux pinning by barium stannate nanoparticles in MOD YBCO coated conductors. *IEEE Trans. Appl. Supercon*, **19**, 3140-3143 (2009).

[94] Birlik, I., Erbe, M., Freudenberg, T., Celik, E., Schultz, L., & Holzapfel, B. Flux pinning improvement of YBCO superconducting films with $BaZrO_3$ nanoparticles prepared by chemical solution deposition method. *J. Phys.: Conf. Series* **234** 012004 (2010).

[95] Lu, F., Kametani, F., & Hellstrom, E.E. Film growth of $BaZrO_3$-doped $YBa_2Cu_3O_{7-\delta}$ by using fluorine-free metalorganic deposition. *Supercond. Sci. Technol.* **25** 015011 (2012).

[96] Haruta, M., Saura, K., Fujita, N., Ogura, Y., Ichinose, A., Maeda, T., & Horii, S. Relationship between vortex pinning properties and microstructure in BaNbO-doped $YBa_2Cu_3O_y$ and $ErBa_2Cu_3O_y$ films. *Physica* C **494** 158-162 (2013).

[97] Poole, C.P., Farach, H.A., Creswick, R.J., Prozorov, R. *Superconductivity* p. 141, 154 (Academic Press, London, 2007).
